# Copper-Deficiency in *Brassica napus* Induces Copper Remobilization, Molybdenum Accumulation and Modification of the Expression of Chloroplastic Proteins

**DOI:** 10.1371/journal.pone.0109889

**Published:** 2014-10-15

**Authors:** Vincent Billard, Alain Ourry, Anne Maillard, Maria Garnica, Laurent Coquet, Thierry Jouenne, Florence Cruz, José-Maria Garcia-Mina, Jean-Claude Yvin, Philippe Etienne

**Affiliations:** 1 Normandie Université, Caen, France; 2 UNICAEN, UMR 950 Ecophysiologie Végétale, Agronomie et nutritions N, C, S, Caen, France; 3 INRA, UMR 950 Ecophysiologie Végétale, Agronomie et nutritions N, C, S, Caen, France; 4 Timac Agro Spain, Poligono de Arazuri-Orcoyen, Orcoyen, Spain; 5 Plateforme de protéomique PISSARO, UMR6270 CNRS Faculté des Sciences de Rouen, Mont-Saint-Aignan, France; 6 Centre de Recherche International en Agroscience, CRIAS-TAI, Groupe Roullier, Dinard, France; Auburn University, United States of America

## Abstract

During the last 40 years, crop breeding has strongly increased yields but has had adverse effects on the content of micronutrients, such as Fe, Mg, Zn and Cu, in edible products despite their sufficient supply in most soils. This suggests that micronutrient remobilization to edible tissues has been negatively selected. As a consequence, the aim of this work was to quantify the remobilization of Cu in leaves of *Brassica napus* L. during Cu deficiency and to identify the main metabolic processes that were affected so that improvements can be achieved in the future. While Cu deficiency reduced oilseed rape growth by less than 19% compared to control plants, Cu content in old leaves decreased by 61.4%, thus demonstrating a remobilization process between leaves. Cu deficiency also triggered an increase in Cu transporter expression in roots (*COPT2*) and leaves (*HMA1*), and more surprisingly, the induction of the *MOT1* gene encoding a molybdenum transporter associated with a strong increase in molybdenum (Mo) uptake. Proteomic analysis of leaves revealed 33 proteins differentially regulated by Cu deficiency, among which more than half were located in chloroplasts. Eleven differentially expressed proteins are known to require Cu for their synthesis and/or activity. Enzymes that were located directly upstream or downstream of Cu-dependent enzymes were also differentially expressed. The overall results are then discussed in relation to remobilization of Cu, the interaction between Mo and Cu that occurs through the synthesis pathway of Mo cofactor, and finally their putative regulation within the Calvin cycle and the chloroplastic electron transport chain.

## Introduction

While N, P, K and more recently S fertilizations are common practices in agriculture, the supply of micronutrients is less frequently considered. However, according to the World Health Organization [1], two billion people around the world suffer from micronutrient deficiencies (mostly Fe, Cu, Zn and Mg), causing 7.3% of disease burden. Among the micronutrients involved in human metabolism, Cu deficiency, for example, causes symptoms of diseases such as immune defects or anaemia [2]. Without stock or sufficient intake, undernourished children are the first affected [3], however Cu deficiency is also observed in developed countries [4],[5]. In the human diet, Cu is mainly present in meat (especially liver and offal), fish and nuts [6]. However, these food supplies are not available to most populations suffering from micronutrients deficiencies. As a consequence, staple foods (cereals, tubers and oils) must be fortified in order to provide the minimum dose of essential micronutrients, such as Cu, for these populations [7].

Over the past 60 years, the micronutrient content (mostly Fe, Zn, Mg and Cu) has been reduced in edible products despite sufficient micronutrient availability in most cultivated soils, and this is the result of varietal selection that aimed to achieve higher yields [8], [9]. Consequently, whether a genetic improvement of micronutrient uptake by roots would increase the edible plant content remains questionable. Alternatively, improving the transfer of such micronutrients in edible parts from remobilization of vegetative tissue could be an alternative for breeding if we assume that the recycling (i.e. remobilization) of such nutrients is a significant process. In addition, it has been shown that some soils can lack Cu resulting in dramatic agricultural effects [10]. Thus, Cu deficiency in plants affects human health either directly (decrease in yield) or indirectly (symptoms induced by a lack of micronutrients) [11], [12].

As a transition metal [13], Cu is involved in numerous processes in plants. Most of Cu’s functions in plants rely on enzymatically bound Cu (more than one hundred proteins identified so far [14]), mostly catalysing redox reactions. As a consequence, only 2% of plant Cu occurs in its free form. Chloroplasts contain about half of the total plant Cu, where it is especially associated with proteins involved in the electron transport chain such as plastocyanin [15]. Three Cu ions are also required for assembly of the active cytochrome c oxydase complex in the mitochondrial electron transport chain. Additionally, numerous proteins known to bind Cu or to be regulated by Cu are involved in the Calvin cycle or in the Tricarboxylic Acid Cycle (TCA), for example Fructose 1–6 Bis Phosphatase (FBPase), Glyceraldehyde 3 Phosphate DeHydrogenase (G3PDH) [16] and PhosphoGlycerate Kinase (PGK) [17]. Cu is also important for enzymes involved in cell detoxification, such as glutathione-S-transferase [18] or Cu-Zn superoxide dismutase [19], or in cell wall metabolism such as ascorbate and polyphenol oxidases [20].

From a genetic point of view, some studies have reported a high variation in Cu content between accessions of the same species such as spinach, pea [21] and cassava [22]. While it has been suggested that Cu management should be easily improved by breeding [4], mechanisms that could increase the Cu content in edible tissues, such as remobilization, have been poorly described in the literature.

An important agricultural crop, such as *Brassica napus*, usually requires high levels of nutrients and hence fertilization to reach an optimal yield with preserved quality. Indeed, winter oilseed rape is highly sensitive to sulphur (S) and nitrogen (N) deficiencies with negative consequences for yield and seed quality [23] and therefore, requires high doses of N and S fertilizers. These strong needs for fertilizers are partly due to the low (macro)-nutrient use efficiency (defined by the ratio of seed to plant nutrient contents) of oilseed rape. For example, it has been reported that its low N use efficiency results from a relatively inefficient endogenous N mobilization [24], [25] that occurs mostly during leaf senescence [26]. In contrast, S remobilization from leaves to the seeds (linked to induction of tonoplastic sulphate transporters allowing the mobilization of sulphate previously stored in leaves [27]) is not triggered by leaf senescence but rather by S deficiency. Therefore, without S deficiency, the S content in fallen leaves remains high, up to 2% of dry weight. According to these studies, *Brassica napus* constitutes an important model of defective agro-environmental interactions in studies of nutrient remobilization from leaves. Moreover, it constitutes a relevant plant species model because its genetic proximity to *Arabidospis thaliana* provides easy access to molecular tools while maintaining an agronomic relevance.

To date, deciphering of the plant response to Cu deficiency has been performed through Fe and Zn interactions, identification and characterization of genes encoding transporters (such as *COPT* and *HMA* families), and monitoring target proteins and metabolites [28]–[31]. However, to our knowledge, the effect of Cu deficiency on growth and plant metabolism is currently poorly reported. Indeed, only one article [32] describes the use of a microarray approach to study modifications of the *Arabidopsis thaliana* transcriptome in response to Cu deficiency.

The first aim of this study was to quantify the effect of Cu deficiency on growth of *Brassica napus* and to assess the mobility of Cu between plant tissues (i.e. Cu remobilization). Moreover, a wider analysis of plant nutrients has been used to monitor the effect of Cu-deficiency on the uptake of some macro (Ca, K, Mg, N, P and S) and micronutrients (B, Fe, Mn, Mo, Na and Zn). Finally, a molecular approach combining proteomic and targeted transcriptomic studies has been used to identify the main metabolic pathways affected by Cu deficiency.

## Materials and Methods

### Growth conditions

Seeds of *Brassica napus* var. Boheme were surface-sterilized by exposure to 80% ethanol for 30 s followed by 20% sodium hypochlorite for 10 min. After 10 washes with demineralised water, seeds were germinated on perlite over demineralised water for 2 days in the dark and 1 week under natural light in a greenhouse. Just after first leaf emergence, seedlings were transferred to a 20 L tank containing the following nutrient solution: KNO_3_ 1.25 mM, Ca(NO_3_)_2_ 1.25 mM, KH_2_PO_4_ 0.25 mM, MgSO_4_ 0.5 mM, EDTA, 2 NaFe 0.2 mM, H_3_BO_3_ 0.01 mM, MnSO_4_ 5 µM, ZnSO_4_ 3 µM, (NH_4_)_6_Mo_7_O_24_ 0.7 µM, CoCl_2_ 0.1 µM, NiCl_2_ 0.04 µM, SiO_2_ 0.1 mM, CaCl_2_ 20 1.25 mM, KCl 0.25 mM. Control nutrient solution also contained NaOH 0.1 mM and CuSO_4_ 0.7 µM while Na_2_SO_4_ 1 µM was added to Cu-depleted nutrient solution. These nutrient solutions were renewed every two days. Plants were grown under greenhouse conditions with a thermoperiod of 20°C/17°C day/night and a photoperiod of 16 h. Natural light was supplemented with high pressure sodium lamps (Philips, MASTER GreenPower T400W) supplying an average photosynthetically active radiation of 280 µmol photons.m^−2^.s^−1^ at canopy height. After one week of growth, plants were separated into 2 sets: control plants receiving normal nutrient solution and Cu-deficient plants receiving the Cu-depleted nutrient solution (for details, see above) over 25 days. Four independent samples, each consisting of three plants, were harvested at the beginning of Cu depletion (t = 0) and after 25 days with (control) or without Cu (–Cu). Leaves and petioles present at the beginning of Cu depletion (referred as “old leaves” and “old petioles”, respectively) were distinguished from leaves appearing during Cu depletion (referred as “young leaves” and “young petioles”, respectively). At each date of harvest (t = 0 and t = 25 days), whole roots from control and Cu-depleted plants were collected. An aliquot of each tissue was weighed and dried in an oven (60°C) for dry weight (DW) determination and ground to fine powder for IRMS and ICP-OES analysis. Thereafter, the remaining fresh tissues were frozen in liquid nitrogen and stored at –80°C for transcriptomic and proteomic analyses.

Every five days throughout the culture period, non-destructive determination of chlorophyll content of young and old leaves was performed using a SPAD chlorophyll meter (SPAD-502 model, Minolta, Tokyo, Japan). The determination was carried using three replicates of ten measurements performed on independent leaves.

### Analysis of nutrients in plant tissues

For the analysis of total N and S contents, an aliquot of around 4 mg DW of each plant organ sample was placed in tin capsules for total N and S analysis using an IRMS spectrometer (Isoprime, GV Instrument, Manchester, UK) linked to a C/N/S analyser (EA3000, Euro Vector, Milan, Italy). The total amount of N or S (N_tot_ or S_tot_) in a tissue “i” at a given time “t” is calculated:
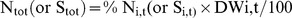



Other nutrients (K, Ca, S, P, Mg, Fe, Na, Mn, B, Si, Zn and Cu) were analysed by Inductively Coupled Plasma Optical Emission Spectrometry (ICP-OES, Thermo Elemental Co. Iris Intrepid II XDL) with prior microwave acid sample digestion (8 mL of concentrated HNO_3_ and 2 mL of H_2_O_2_ for 0.5 g DW), using a protocol previously described by Mora *et al.* [33].

### RNA extraction

Total RNA was extracted from 200 mg of frozen samples ground to a powder with a pestle in a mortar containing liquid nitrogen. The resulting powder was suspended in 750 µl of extraction buffer [0.1 M TRIS, 0.1 M LiCl, 0.01 M EDTA, 1% SDS (w/v), pH 8] and 750 µl of hot phenol (80°C, pH 4). This mixture was vortexed for 30 s and, after addition of 750 µl of chloroform/isoamylalcohol (24∶1, v/v), the homogenate was centrifuged (15 000 g, 5 min, 4°C). The supernatant was transferred into a 4 M LiCl solution and incubated overnight at 4°C. After centrifugation (15 000 g, 30 min, 4°C), the pellet was suspended in 100 µl of sterile water. RNA was then purified with an RNeasy mini kit according to the manufacturer’s protocol (Qiagen, Courtaboeuf, France). Quantification of total RNA was performed by spectrophotometry at 260 nm (BioPhotometer, Eppendorf, Le Pecq, France) before Reverse Transcription (RT) and real time Quantitative Polymerase Chain Reaction (Q-PCR) analysis.

### Reverse transcription (RT) and Q-PCR analysis

For RT, 1 µg of total RNA was converted to cDNA with an “iScript cDNA synthesis kit” according to the manufacturer’s protocol (Bio-Rad, Marne-la-Coquette, France).

Q-PCR amplifications were performed using specific primers for each housekeeping gene (*EF1-α* and *18S rRNA*) and target genes (Table 1). Q-PCRs were performed with 4 µl of 200x diluted cDNA, 500 nM of primers, and 1x SYBR Green PCR Master Mix (Bio–Rad, Marne–la–Coquette, France) in a real-time thermocycler (CFX96 Real Time System, Bio–Rad, Marne–la–Coquette, France). A 2 step program, composed of 42 cycles of a denaturing step at 95°C for 15 s followed by an annealing and extending step at 60°C for 40 s, was used for all pairs of primers (Table 1) except for *COPT2*, for which we used a 3 step program. In this case the denaturing step was as described previously. Annealing was at 58.6°C for 10 s and was followed by an extending step at 72°C for 30 s. For each pair of primers, a threshold value and PCR efficiency were determined using a cDNA preparation diluted >10-fold. For all pairs of primers, PCR efficiency was around 100%. The specificity of PCR amplification was examined by monitoring the presence of the single peak in the melting curves after Q-PCRs and by sequencing the Q-PCR product to confirm that the correct amplicon was produced from each pair of primers (Eurofins, Ebersberg, Germany). The relative expression of the genes in each sample was compared with the control sample [corresponding to untreated plants at the same time of harvest] and was determined with the delta delta Ct (ΔΔCt) method using the following equation [34]: Relative expression = 2^−ΔΔCt^, with ΔΔCt = ΔCt_treated_-ΔCt_control_ and with ΔCt  =  Ct_target gene_ – [√(Ct*_EF1_*_−*α*_×Ct*_18S rRNA_*)], where Ct refers to the threshold cycle determined for each gene in the exponential phase of PCR amplification and [√(Ct_EF1−*α*_×Ct*_18S rRNA_*)] corresponds to the geometric average of Ct of the reference genes. Using this analysis method, the relative expression of the different genes in the control sample of the experiment was equal to 1 [34], and on this basis the relative expression of other treatments was then compared with the control. For *COPT2*, because no transcript was detected in control plants (*i.e.* Ct_control_ is undetermined), values are not expressed relatively to control but as 2^−ΔCt^.

**Table 1:** Q-PCR primer sets.

Gene	Accession number	Forward	Reverse	Gene Function
*EF1-α*	DQ312264	5′-gcctggtatggttgtgacct-3′	5′-gaagttagcagcacccttgg-3′	
*18S rRNA*	GQ380689	5′-cggataaccgtagtaattctag-3′	5′-gtactcattccaattaccagac-3′	
COPT2	NM_114557	5′-tgcacatgaccttcttttgg-3′	5′-gtcatcggagggtttgttga-3′	Cu uptake
HMA1	NM_119890.6	5′-gtacagctgaccgaggaagc-3′	5′tgcccataaatgggttcaat-3′	Cu allocation to chloroplast
MOT1	NM_128127	5′-ctcgccaggatttggactta-3′	5′agatccccaacacgaacaag-3′	Mo uptake

*EF1α* and *18S rRNA* were housekeeping genes used for relative gene expressions by Q-PCR analysis.

### Extraction and determination of total proteins

Two hundred mg of fresh matter from old leaf samples were ground to a fine powder in liquid nitrogen in the presence of 50 mg of poly(vinylpolypyrrolidone) (PVPP). The addition of PVPP is used to fix plant polyphenols that might interfere with the quantification of proteins or during separation of proteins by electrophoresis. The ground material was dissolved in 1.75 mL of TCA/acetone solution (10% TCA (w/v) prepared in acetone). After centrifugation (3 min, 16 000 g, 4°C), the protein pellet was purified according to the protocol adapted from Wang *et al.* [35]. The protein pellet obtained after precipitation with TCA/acetone (10% TCA (w/v) was suspended in 1.75 mL of 0.1 M ammonium acetate dissolved in 80% methanol. After homogenization and centrifugation (16 000 g, 3 min, 4°C), the pellet was washed with 1.75 mL of 80% acetone and centrifuged again (16 000 g, 3 min, 4°C). The supernatant was removed and the pellet was dried under vacuum (Speedvac Concentrator 5301, Eppendorf, France) for 5 min at 50°C and then suspended with 0.8 mL of phenol at pH 7.9 and in 0.8 mL of dense SDS buffer (30% sucrose, 2% SDS, 0.1 M Tris–HCl, pH 8.0, 0.5% 2–mercaptoethanol). After 5 min incubation at 4°C and centrifugation (16 000 g, 3 min, 4°C), the phenol phase was transferred to a new tube and supplemented with 1.75 mL of 0.1 M ammonium acetate and stored at –20°C overnight. Afterwards, ammonium acetate was used to precipitate proteins to enable their collection by centrifugation (16 000 g, 5 min, 4°C). The protein pellet was then washed with 1.75 mL of 100% methanol and again with 1.75 mL of 80% acetone. Residual acetone was removed by vacuum evaporation over a few minutes. The pellet was resuspended in 400 µL of R2D2 rehydration buffer [5 M urea, 2 M thiourea, 2% CHAPS, 2% N–decyl–N,N–dimethyl–3–ammonio–1–propanesulfonate, 20 mM dithiothreitol, 5 mM Tris (2–carboxy– ethyl) phosphine, 0.5% IPG buffer] (GE Healthcare, Saclay, France), pH 4 to 7 [36]. The total protein concentration was determined by the method previously described by Bradford [37] using bovine serum albumin as standard.

### Two–dimensional electrophoresis (2–DE) and image analysis

2–DE was performed according to the protocol detailed by Desclos *et al*. [25]. Gels were stained using the silver–staining procedure described by Blum *et al.* [38] and scanned with a ProXPRESS 2D proteomic imaging system (Perkin–Elmer, Courtaboeuf, France). Images of the 2–DE gels were analysed using the Progenesis SameSpots software v3.0 (Nonlinear Dynamics, Newcastle upon Tyne, UK) according to the manufacturer’s protocol. Gels from three independent biological replicates were used. Spot detection, warping, and matching were performed automatically by the software and manually validated. Artefacts due to non–specific silver nitrate staining or spots that could not be confidently verified as true matches were disregarded rather than manually edited, and misalignments were corrected by manual warping when appropriate. Mw and *pI* were calculated using SameSpots software calibrated with commercial molecular mass standards (prestained precision protein standards; Bio–Rad, Marne–la–Coquette, France) run in a separate marker lane on 2–DE gel.

### RuBisCO relative quantification

In order to observe maximum spots, silver staining was performed on 2-DE with a saturated RuBisCO signal. The amount of RuBisCO was then measured by Experion Pro260 Analysis Kit (Bio–Rad, Marne–la–Coquette, France). The Experion automated electrophoresis system applies a combination of microfluidic separation technology and sensitive fluorescent detection of proteins [39]. The protein extracts were treated with the Experion reagents and then separated on the Experion automated electrophoresis station according to the manufacturer’s instruction. Peak areas corresponding to large and small RuBisCO subunits were then compared using software given by manufacturer.

### Protein Identification by ESI LC–MS/MS

Spots of interest were excised from 2D gels and washed several times with water and dried for a few minutes. Trypsin digestion was performed overnight with a dedicated automated system (MultiPROBE II, Perkin-Elmer). The gel fragments were subsequently incubated twice for 15 min in a 0.1% CH_3_CN solution in water to allow extraction of peptides from the gel pieces. Peptide extracts were then dried and dissolved in starting buffer for chromatographic elution, consisting of 3% CH_3_CN and 0.1% HCOOH in water. Peptides were enriched and separated using lab–on–a–chip technology (Agilent, Massy, France) and fragmented using an on–line XCT mass spectrometer (Agilent). The ESI LC–MS/MS data were converted into DTA– format files that were further searched for proteins with MASCOT Daemon (Matrix Science [40]). For protein identification, two strategies were employed to mine the maximum data. Measured peptides were searched in the NCBI nr–protein sequence database, *viridiplantae* (green plants [41]), and in the *Brassica* EST database (*Brassica* Genome Gateway 2007, [42]). Proteins with two or more unique peptides matching the protein sequence with a score >53, as defined by MASCOT, were considered as a positive identification. The spectra of each peptide were verified manually. In cases where protein identification data were lacking, BLAST analysis was performed [43].

### Data and statistical analysis

Regarding the growth, ICP-OES and IRMS analyses, all experiments were conducted with 4 independent biological replicates each corresponding to 3 plants. Proteomics and Q-PCR analyses were performed on 3 independent biological replicates each corresponding to 3 plants. Data are given as mean ± SE for n = 3 or 4. For Q-PCR, SE and Student’s T-test were based on ΔΔCt (or ΔCt for *COPT2*, see above for more information), when the relation between values is still linear. According to Yuan *et al.* 2006 [44], a confidence interval of each mean value (with min and max values) can be calculated and has been presented in the results section as [mean of relative expression] (Min value–Max value) with Min value = 2^−(ΔΔCt+SE)^ and Max value = 2^−(ΔΔCt−SE)^ (Min value = 2^−(ΔCt+SE)^ and Max value = 2^−(ΔCt−SE)^ for *COPT2*). Conversion of the results of an exponential process into a linear comparison of amounts leads to an asymmetric distribution [34].

All data were analysed with Student’s T-test (p<0.05) and marked by an asterisk (*) or cross (†) when significantly different.

## Results

### Cu depletion affects the growth and Cu content of *B. napus*

After 25 days of culture, control whole plant DW increased from 3.23±0.24 g to 39.67±1.26 g (table 2). The total DW of Cu-depleted plants was decreased by 18.4% relative to control plants (table 2, from 39.67 to 32.37 g DW.plant^−1^). This was the result of a significant reduction in root DW (from 4.11±0.23 in control plants to 3.73±0.06 g DW.plant^−1^ in Cu-depleted plants) and secondly, of old petioles DW (from 8.07±0.60 to 6.31±0.49 g DW.plant^−1^). No significant differences in growth rates were found for other organs. Control plants accumulated 251.5 µg of Cu while the Cu content in Cu-depleted plants only rose from 29.0±4.0 µg to 74.8±3.0 µg. The slight increase in total Cu in Cu-depleted plants was the result of a trace of Cu found in the mineralized water used for the nutrient solution (0.47±0.00 µM, data not shown). In roots of control plants, the amount of Cu increased from 14.7±3.0 µg to 155.2±13.2 µg but only reached 43.4±1.5 µg in Cu deficient plants. In aerial tissues that developed under Cu depletion, the Cu amount was reduced from 11.8±1.9 µg to 2.6±0.7 µg and from 76.2±4.8 µg to 16.2±2.3 µg in petioles and leaves, respectively. In petioles already present at the time of the Cu depletion, the amount of Cu rose from 2.1±0.2 µg to 8.0±0.7 µg during depletion while it reached 20.3±2.4 µg in control plants at the end of the experiment. The amount of Cu in old leaves of control plants did not vary significantly during the experiment, while in Cu-depleted plants, it was reduced from 12.2±1.1 µg to 4.7±1.0 µg indicating a remobilization of 61.4±4.2% of the Cu initially present in these leaves.

**Table 2:** Biomass and Cu content in *Brassica napus* L. at t = 0 and after 25 days of culture with (control) or without Cu (−Cu).

		t = 0	Control (25d)	-Cu (25d)
Old leaves	DW (g)	2.13±0.17	4.78±0.79	4.83±0.93
	Cu amount (µg)	12.2±1.1	17.0±2.5	4.7±1.0*†
	% remobilized	NA	NA	60.36±4,15
Old petioles	DW (g)	0.63±0.04	8.07±0.60	6.31±0.49*†
	Cu amount (µg)	2.1±0.2	20.3±2.4*	8.0±0.7*†
Young leaves	DW (g)	NA	12.7±0.94	8.83±1.64
	Cu amount (µg)	NA	76.2±4.8	16.2±2.3 †
Young petioles	DW (g)	NA	10.02±0.78	8.66±0.55
	Cu amount (µg)	NA	11.8±1.9	2.6±0.7 †
Roots	DW (g)	0.48±0.03	4.11±0.23*	3.73±0.06*
	Cu amount (µg)	14.7±3.0	155.2±13.2*	43.4±1.5*†
Total	DW (g)	3.23±0.24	39.67±1.26*	32.37±1.71*†
	Cu amount (µg)	29.0±4.0	280.5±13.5*	74.8±3.0*†

* and † represent significant differences at p<0.05 compared to t = 0 and control, respectively. NA: Not Applicable.

**Figure 1:**
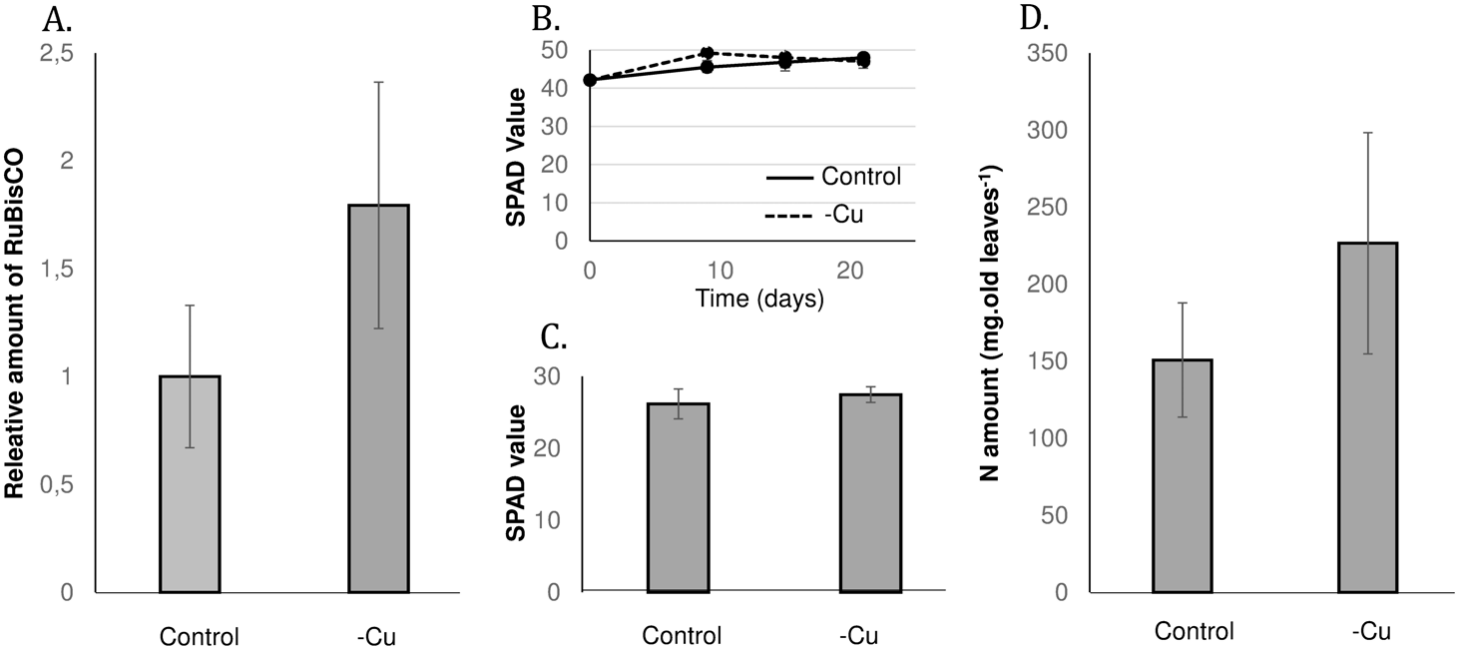
Parameters associated with senescence. (A.) Relative amount of RuBisCO in old leaves. Relative chlorophyll content (SPAD value) during (B.) the whole experiment at whole plant level and (C.) after 25 days of depletion in old leaves (D.) N content in old leaves.

No significant difference was observed for RuBisCO by *Experion* method (Figure 1.A). During the whole experiment, the leaf chlorophyll content (SPAD values, Figure 1.B) were not significantly different between control and Cu-depleted plants. At the end of the experiment, this was confirmed in old leaves (Figure 1.C). Also, N content in old leaves didn’t present any difference between the two conditions (Figure 1.D).

### Cu depletion increases expression of Cu transporters in *Brassica napus*

**Figure 2:**
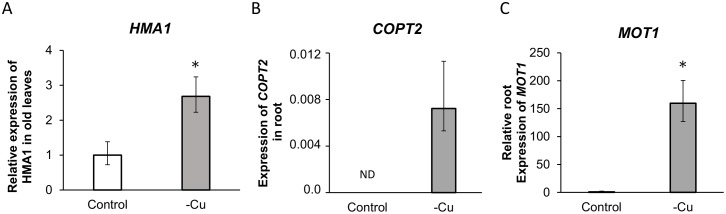
Relative accumulation of transcript of Cu transporters (A. *HMA1* in old leaves and B. *COPT2* in roots) and Molybdenum transporter 1 (C. *MOT1* in roots) in *Brassica napus* L after 25 days of culture with (control) or without Cu (−Cu). Control is represented by white bars and –Cu by grey bars. *MOT1* and *HMA1* values are expressed as 2^−ΔΔCt^ while *COPT2 value* is expressed as 2^−ΔCt^ (for details, see “Material and Methods”). * represents significant differences at p<0.05. ND: not detected.

The relative expression of genes involved in Cu transport (*HMA1, COPT2*) is given in Figure 1. Expression of the *HMA1* gene encoding a chloroplastic Cu transporter was monitored in leaves, whereas *COPT2*, which encodes a transporter involved in Cu uptake, was monitored in roots. The relative number of *HMA1* transcripts increased 2.68 fold (2.23–3.24) after 25d of Cu depletion (Figure 2.A). Transcripts of *COPT2* were detected only in roots from Cu-depleted plants suggesting that the expression of the *COPT2* transporter was up-regulated and/or de-repressed in *Brassica napus* roots in response to Cu deficiency (Figure 2.B).

### Cu depletion affects uptake of some mineral nutrients

**Figure 3:**
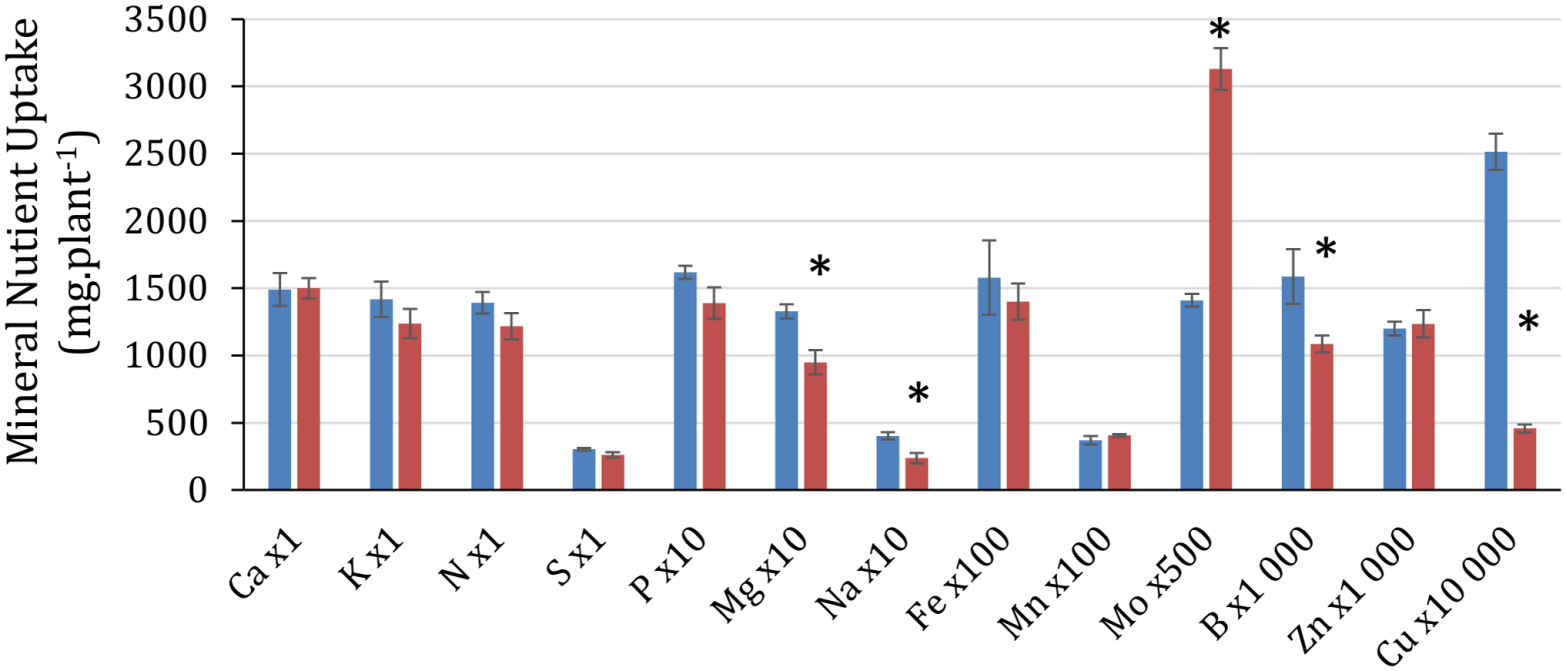
Mineral nutrient uptake in control plants (blue) and Cu-depleted plants over 25 Days (red). Due to large differences between nutrients, factors have been attributed to each nutrient to allow representation on the same graph. * indicates significant difference between control and Cu depleted plants at p = 0.05.

Figure 3 shows that Cu deprivation decreased Cu uptake by 81.8±2.0%, but also affected the uptake of Na (−41.0±3.8%), B (−31.6±2.1%) and Mg (−28.5±2.8%). while plant dry weight was reduced by 18.4±2.5% (Table 2). Cu deficiency had no effect on total plant uptake for nutrients such as N, Ca, K, S, P, B, Fe, Mn and Zn, despite the difference of biomass. In contrast, uptake of Mo was increased by 121.8% despite the reduced plant growth and was associated with a very strong up-regulation of the molybdenum transporter 1 gene (*MOT1,* Figure 2.C), having an important role in efficient Mo uptake [45], [46]. Indeed, in Cu-depleted plants, the number of *MOT1* transcripts was found to be 159.61 fold (127–200) higher than in control plants.

### Cu depletion induces proteomic modifications

**Figure 4:**
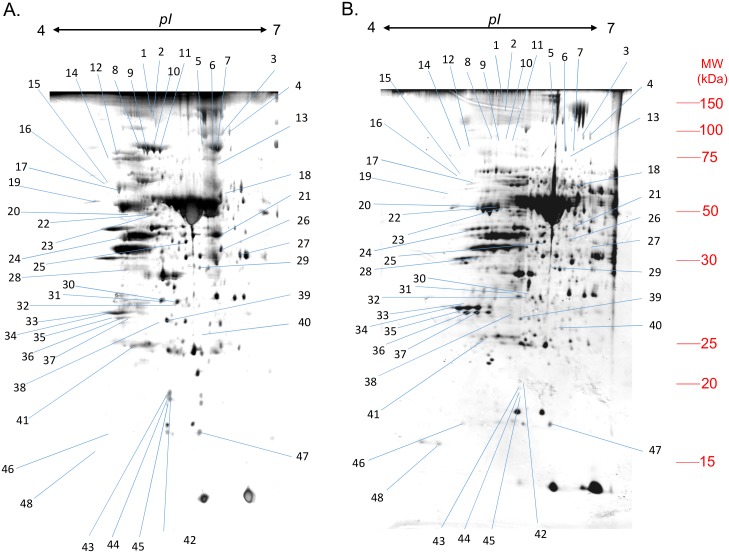
Representative Silver-stained 2-DE gels of proteins from old leaves of *Brassica napus* L. after 25 days of culture with (control, A) or without Cu (−Cu, B). A total of 150 µg of total proteins was loaded on an IEF strip forming an immobilized pH linear gradient from 4 to 7 (for details, see ‘‘Materials and Methods’’). Numbers indicate sequenced and identified proteins presented in table 3. Molecular mass markers (MW in kDa) are listed on the right side of the gel.

Leaves already present at the beginning of Cu depletion were the most affected tissues, not only due to their decreased Cu content and hence the mobilization of Cu (by about 60%) to other tissues, but also according to their decrease of Mg (−42.1±11.0%), Na (−42.6±13.6%), Ca (−35.8±10.0%) and Fe (−27.9±13.5%) contents. Therefore, leaves were selected for a comparative proteomic approach in order to highlight the main metabolic modifications induced by Cu depletion. Representative 2D gels corresponding to protein extracts from leaves of control and Cu-depleted plants are shown in Figure 4. On each gel, 693 proteins were identified. Their comparative analysis revealed 48 proteins significantly up- or down-regulated by Cu depletion. Because different spots correspond to different isoforms of the same protein, only 33 different proteins were identified. Among them, 25 proteins were accumulated in Cu-depleted old leaves. Transketolase-like protein (#7 to 11, Figure 4 and Table 3) was the strongest protein induced with four isoforms that were accumulated more than fourfold compared to control. On the other hand, repression was significant in only 8 of the 33 identified proteins, with the Oxygen Evolving Enhancer (OEE, #33 to 37) having the greatest repression in our study (−2.3 to −3.93). Among all proteins (Table 3), five were involved in stress responses (disease/defence or defence/secondary metabolite), for example glutathione reductase (#18), and seventeen proteins were involved in energy processes, for example aldolase (#27) and ATP synthase (#17). Protein annotations in databases also indicated that chloroplasts were the main locations of the identified proteins with 17 references, including the most repressed, OEE (#33 to 37), and the most induced, Transketolase-like protein (#7 to 11).

**Table 3:** Abundance of identified proteins over (positive value) or under (negative value) accumulated in old leaves of Cu-depleted *B. napus* relative to control plants.

Spot no.	Relative Abundance	Exp. pI/Mw	Theo. pI/Mw	PM	SC (%)	Protein name/Organism/NCBI accession no.	Functional classification/Sub-Cellular localization	Note
1	3.12	5.7/115	5.39/104.3	30	20	Myrosinase binding protein [*Brassica napus*]/gi|1711296	11. Disease/Defence	
2	2.11	6.3/99	6.59/102.2	117	40	Chaperone protein ClpC, chloroplastic-like [*Fragaria vesca* subsp. vesca]/gi|470122943	08. Intracellular traffic/Chloroplast	
3	1.75	6.64/90	6.12/84.3	58	24	Cobalamin-independent methionine synthase [*Arabidopsis lyrata* subsp. lyrata]/gi|297807807	01. Metabolism	**Zn** [73]
4	2.78	6.53/91	6.12/84.3	29	19	Cobalamin-independent methionine synthase [*Arabidopsis lyrata* subsp. lyrata]/gi|297807807	01. Metabolism	**Zn** [73]
5	4.16	6.26/80	6.16/81.2	13	13	NADH Ubiquinone oxydoreductase 75 kD subunit [*Arabidopsis thaliana*]/gi|30693102	02. Energy/Chloroplast-Mitochondria	[Fe-S] cluster
6	5.23	6.5/81	5.80/81.5	15	18	Transketolase-like protein [*Arabidopsis thaliana*]/gi|7329685	02. Energy/Chloroplast-Mitochondria	
7	2.22	6.41/80	6.12/79.9	13	10	Transketolase [*Arabidopsis thaliana*]/gi|30689983	02. Energy/Chloroplast-Mitochondria	
8	4.79	5.74/75	6.16/81.2	46	24	Transketolase-like protein [*Arabidopsis thaliana*]/gi|7329685	02. Energy/Chloroplast-Mitochondria	
9	4.17	5.78/75	6.16/81.2	61	27	Transketolase-like protein [*Arabidopsis thaliana*]/gi|7329685	02. Energy/Chloroplast-Mitochondria	
10	4.46	5.67/76	6.16/81.2	62	29	Transketolase-like protein [*Arabidopsis thaliana*]/gi|7329685	02. Energy/Chloroplast-Mitochondria	
11	4.23	5.54/74	6.16/81.2	44	26	Transketolase-like protein [*Arabidopsis thaliana*]/gi|7329685	02. Energy/Chloroplast-Mitochondria	
12	2.06	5.25/73	5.08/70.7	49	37	Heat shock cognate protein HSC70 [*Brassica napus*]/gi|2655420	08, intracellular traffic/Chloroplast	*Zn* [16]
13	−2.07	6.48/70	5.56/63.4	10	11	Phosphoglucomutase [*Arabidopsis thaliana*]/gi|15223226	02. Energy/Chloroplast	
14	−2.03	5.17/69	5.62/72.0	25	16	ATP-dependent zinc metalloprotease FTSH 2, chloroplastic-like [*Brachypodium distachyon*]/gi|357123383	06. Prot. Dest. & Storage/Chloroplast	***Zn*** [74]
15	1.54	6.14/64	5.22/49.4	11	7	Thylakoid rhodanese-like protein [*Arabidopsis thaliana*]/gi|18411523	02. Energy/Chloroplast	***Cu*** and ***Zn*** [75]
16	1.4	6.08/62	5.66/47.6	5	4	Thylakoid rhodanese-like protein [*Arabidopsis thaliana*]/gi|18411523	02. Energy/Chloroplast	***Cu*** and ***Zn*** [75]
17	2.63	5.25/62	5.97/47.0	85	42	ATPase subunit I [*Arabidopsis lyrata* subsp. lyrata]/gi|297837977	02. Energy/Chloroplast-Mitochondria	*Cu* and *Zn* [16]
18	−2.67	6.52/61	8.03/60.6	15	19	Glutathione reductase [*Brassica juncea*]/gi|13448672	20. Defence & Secondary metabolism/Vacuole	*Cu* [16]
19	2.09	4.92/55	5.23/50.6	7	5	Rhodanese-like domain-containing protein 4, chloroplastic-like [*Cicer arietinum*]/gi|502179307	01. Metabolism/Chloroplast	***Cu*** [75]
20	−2.27	5.45/50	6.49/51.0	19	16	NAD(P)H dehydrogenase subunit 48 [*Arabidopsis thaliana*] gi|18394307	02. Energy/Chloroplast	
21	1.52	6.44/47	5.91/50.1	25	21	Phosphoglycerate kinase 1 [*Arabidopsis thaliana*]/gi|15230595	02. Energy/Chloroplast-Mitochondria	*Cu* [17]
22	1.92	5.58/53	6.06/54.2	19	19	Mitochondrial processing peptidase alpha subunit, putative [*Arabidopsis thaliana*] gi|21594004	06. Prot. Dest. & Storage/Mitochondria	
23	2.81	5.53/52	6.06/54.2	7	11	Mitochondrial processing peptidase alpha subunit, putative [*Arabidopsis thaliana*] gi|21594004	06. Prot. Dest. & Storage/Mitochondria	
24	2.97	5.11/46	6.78/47.9	61	46	Chloroplast ribulose-1,5-bisphosphate carboxylase/oxygenase activase [*Brassica oleracea*]/gi|383470439	02. Energy/Chloroplast	
25	1.7	6.03/44	6.43/50.2	44	32	Glutamate-1-semialdehyde 2,1-aminomutase 2 [*Arabidopsis thaliana*]/gi|15229018	01. Metabolism	B6 dependent [56]
26	1.42	6.44/42	5.91/15.1	16	45	Cinnamyl alcohol dehydrogenase [*Brassica napus*]/gi|6683959	01. Metabolism	
27	1.46	6.64/40	6.22/38.4	19	37	Fructose-bisphosphate aldolase [*Arabidopsis thaliana*]/gi|15231715	02. Energy/Chloroplast-Mitochondria	*Cu* [16] and ***Zn*** [76]
28	1.47	5.24/37	5.14/32.0	9	35	40S ribosomal protein [*Brassica napus*]/gi|15214300	05. Protein synthesis/Ribosome	
29	1.33	6.16/37	8.54/35.8	29	31	Mitochondrial NAD-dependent malate dehydrogenase [*Arabidopsis thaliana*]/gi|21592905	02. Energy/Mitochondria	*Cu* [16] and ***Zn*** [77]
30	1.72	5.7/32	8.32/40.3	49	34	Ferredoxin-NADP(+)-oxidoreductase 1 [*Arabidopsis thaliana*]/gi|15239282	02. Energy/Chloroplast	[Fe-S] cluster
31	1.56	5.8/32	8.66/40.1	34	29	Ferredoxin-NADP+ reductase [*Arabidopsis thaliana*]/gi|5730139	02. Energy/Chloroplast	[Fe-S] cluster
32	2.1	5.84/32	6.12/33.0	13	25	Pyridoxine biosynthesis protein [*Lotus japonicus*]/gi|72256517	11. Disease/Defence/Cytosol	B6 biosynthesis [55]
33	−3.93	4.88/31	5.92/35.0	5	15	Oxygen-evolving enhancer protein 1–2 [*Arabidopsis thaliana*]/gi|15230324	02. Energy/Chloroplast	***Mn*** [78]
34	−2.3	4.92/30	5.92/35.0	81	54	Oxygen-evolving enhancer protein 1–2 [*Arabidopsis thaliana*]/gi|15230324	02. Energy/Chloroplast	***Mn*** [78]
35	−2.56	4.94/30	5.93/35.1	36	48	Photosystem II subunit O-2 [*Arabidopsis lyrata* subsp. lyrata]/gi|297819782	02. Energy/Chloroplast	***Mn*** [78]
36	−2.95	5.03/30	5.93/35.1	67	52	Photosystem II subunit O-2 [*Arabidopsis lyrata* subsp. lyrata]/gi|297819782	02. Energy/Chloroplast	***Mn*** [78]
37	−2.8	5.32/30	5.92/35.0	84	55	Oxygen-evolving enhancer protein 1–2 [*Arabidopsis thaliana*]/gi|15230324	02. Energy/Chloroplast	***Mn*** [78]
38	1.69	5.61/29	8.65/33.7	13	11	Thioredoxin-like protein CDSP32 [*Arabidopsis thaliana*]/gi|15222954	11. Disease/Defence/Cytosol	
39	3.81	5.73/29	5.54/29.5	23	17	Carbonic anhydrase 1 [*Arabidopsis thaliana*]/gi|30678347	01. Metabolism/Chloroplast	***Cu*** and ***Zn*** [79]
40	−1.81	6.17/27	6.28/27.6	29	34	Mitochondrial F1F0-ATP synthase subunit Fad [*Arabidopsis thaliana*]/gi|15227104	02. Energy/Mitochondria	Cu [18] and *Zn* [16]
41	1.55	5.28/26	7.67/33.3	10	16	Triosephosphate isomerase [*Arabidopsis thaliana*]/gi|15226479	02. Energy/Chloroplast-Mitochondria	
42	2.41	5.73/20	5.37/19.9	9	26	Uncharacterized protein [*Arabidopsis thaliana*]/gi|18391006	13. Unclassified	
43	2.79	5.7/19	5.47/35.7	41	38	Chloroplast beta-carbonic anhydrase [*Brassica napus*]/gi|297787439	01. Metabolism/Chloroplast	***Cu*** and ***Zn*** [79]
44	2.6	5.75/19	6.81/21.8	6	17	Germin-like protein [*Arabidopsis thaliana*]/gi|1755154	12. Unclear Classification	
45	1.99	5.7/19	6.27/22.0	7	23	RecName: Full = Germin-like protein 1; Flags: Precursor [*Sinapis alba*]/gi|1169944	12. Unclear Classification	
46	−2.6	4.99/17	9.12/24.7	18	31	Peroxiredoxin-2E [*Arabidopsis thaliana*]/gi|15231718	11. Disease/Defence/Cytosol	
47	2.74	6.11/17	8.80/24.3	22	22	Rieske FeS protein [*Arabidopsis thaliana*]/gi|9843639	02. Energy/Chloroplast	[Fe-S] cluster
48	−1.76	4.58/16	5.24/17.9	6	15	Probable glycine cleavage system H protein 2 [*Arabidopsis thaliana*] gi|15223217	02. Energy/Chloroplast	

Experimental and theoretical pI/Mw, the number of LC–MS/MS matched peptides (PM), the SCORE and the percentage of sequence coverage (SC) obtained are also indicated. For each protein, the assigned best–matched protein is listed with the organism in which it was identified and its GenBank protein accession number is indicated. Elements given in notes correspond to ligand (in italics) or regulators (in bold) of the corresponding protein described in previous studies (indicated in brackets).

Considering some of the characteristic Cu proteins reported in the literature [47], Plastocyanin was too small (around 10 kDa) to be found on our gels while Cu-Zn superoxide dismutase and Polyphenol oxidase were not differentially expressed and/or detected.

## Discussion

### Cu remobilization from old leaves is induced by Cu deficiency

In this study, the effectiveness of Cu deficiency can be attested by the low Cu concentration found in old and young leaves of Cu depleted plants (0.97±0.03 µg.g^−1^ DW and 1.66±0.40 µg.g^−1^ DW, respectively) which was lower than the usually admitted minimum concentration (5 µg.g^−1^ DW) [48]. Using Cu deficiency we were able to demonstrate a decrease in the Cu content of leaves by 61.4±4.2% (Table 2). To our knowledge, alongside work on *Arabidopsis thaliana* [49], this is one of the few studies that shows that Cu can be recycled between leaves. However, the Cu remobilization could be underestimated in our conditions because some traces of Cu were found in the nutrient solution, as suggested by the slight increase in the overall Cu content in Cu-depleted plants (Table 2). A precise estimation of Cu remobilization would require the use of stable isotopes. This remobilization of Cu in leaves was concomitant with an increase in *COPT2* and *HMA1* expression. Despite a tendency of an increase of RuBisCO and N amounts, the RuBisCO, N amounts and chlorophyll contents in old leaves were not significantly differentially affected between control and Cu-depleted plants (Figure 1). Then we could assume that Cu remobilization was not associated with leaf senescence. This is also consistent with the down-regulation of senescence-associated proteases such as FtsH2 (#14, Table 3), a protease involved in the degradation of photosystem II [26], [50]. Thus, this study suggests that Cu depletion induces a Cu mobilization independent of the distinguishing marks of senescence. This is different to what has been found for N for example, whose deficiency is known to increase leaf senescence in *Brassica napus* [25].

### Effect of Cu deficiency on metabolic pathway in chloroplasts from old leaves of *B. napus*

**Figure 5:**
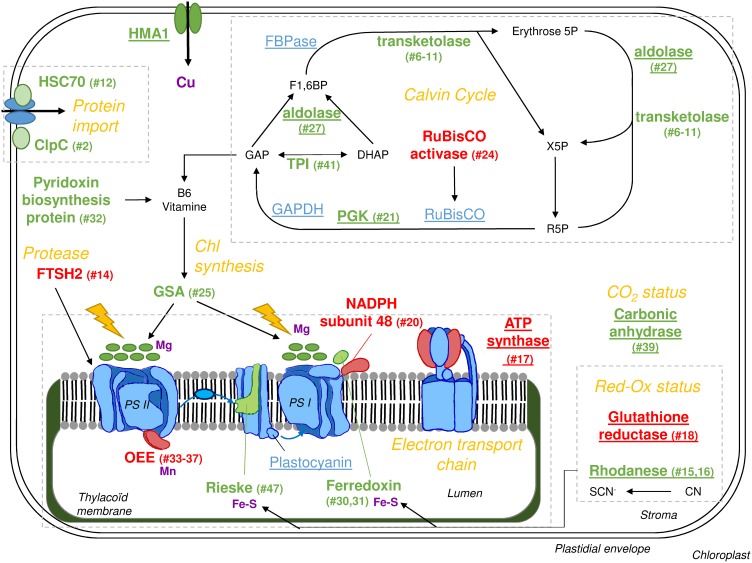
Effect of Cu deficiency on metabolic pathwayspathway in chloroplasts from old leaves of *B. napus*. Bold red indicates a decrease in the amount of protein while bold green indicates an increase. Spot number(s) corresponding to proteins identified in table 3 are indicated in parentheses. Intermediate metabolites are in black, proteins not identified in 2D–E analysis are in blue. Pathways are encircled by grey dashed lines and named in orange italics. Underlines indicate Cu binding and/or known Cu regulation. Other elements known to bind and/or regulate proteins are indicated in purple. PS I: Photosystem I; PS II Photosystem II; R5P: ribulose-5-phosphate; X5P: xylose-5-phosphate; F1,6BP: Fructose-1,6-bisphosphate; GAPDH: GlycerAldehyde Phosphate DeHydrogenase; PGK: PhosphoGlucoKinase; GAP: GlycerAldehyde-3-Phosphate; FBPase: Fructose-1,6-BisPhosphatase; TPI: Triose Phosphate Isomerase; GSA: Glutamate*-*1*-*Semialdehyde 2,1*-*Aminomutase; OEE: Oxygen Evolving Enhancer; Chl: Chlorophyll; CN: cyanate; SCN: Thiocyanate.

Because the spots of our 2D–E gels correspond to the most abundant proteins in old leaves, numerous proteins (especially transporters and transcription factors) corresponding to transcripts identified in previous studies as regulated by Cu depletion could not be observed in the present work. Thus, this study complement previous transcriptomic data on Cu remobilisation [51], [52]. This proteomic analysis of Cu-deficient old leaves showed that more than half of the up or down-regulated proteins were localised in chloroplasts (Table 3) indicating potential disturbance, despite an absence of chlorophyll content variation (Figure 1.B, C). Five of these proteins are involved in the Calvin cycle and 5 are involved in the thylakoid electron transport chain (Figure 5). From a general point of view, among the 33 proteins that were differentially expressed relatively to control plants (Table 3), 11 of them are known to rely on the presence of Cu for their synthesis and/or activity, and 9 were over expressed (#15, 16, 17, 19, 27, 29, 39 and 43). As a consequence, it could be suggested that the lack of Cu reduced their activity which may explain their up-regulation (or alternatively reduced their down-regulation). A similar regulation would also occur for enzymes that are located upstream or downstream of such copper deficiency up-regulated proteins. For example, this is the case (Figure 5) within the Calvin cycle for Transketolases (#6–11) or TPI (#41), which are located upstream and downstream of Aldolase (#27), a copper dependent protein that was up-regulated by Cu-deficiency.

Among Cu binding or regulated enzymes within the Calvin cycle (FBPase [53], GAPDH, Aldolase [16], RuBisCO [18], PGK [17]), only Aldolase (#27) and PGK (#21) were found to be over represented (Table 3 and Figure 5). However, whilst RuBisCO was not significantly affected (Figure 1), its main regulator RuBisCO activase (#24) was increased by about 3 folds (Table 3). It should be noted that PGK, Aldolase, Transketolase and TPI are also involved in glycolysis (in the cytosol or plastids [54]) and their regulation should therefore induce mitochondrial defects.

GAP is a crosspoint between numerous pathways, with B6 vitamin biosynthesis being one of them [55]. However, under an impaired Calvin cycle, more GAP would be available for this cycle (Figure 5). Vitamin B6 can also be synthesized through Pyridoxin biosynthesis protein (#32, Table 3) which is over represented in old leaves of Cu-depleted *B. napus*. The B6 dependent protein Glutamate-1-semialdehyde aminomutase (GSA, #25) [56] with a role in chlorophyll biosynthesis also accumulated (Figure 5).

Differentially expressed proteins located in thylakoids (Figure 5) could be subdivided into two groups: the first one including OEE (#33 to 37), NADPH subunit 48 (#20) and ATP synthase (#17) were down-regulated, which is consistent with previous work reporting a down-regulation of photosynthesis-related genes in response to Cu deficiency [51]. The second group corresponded to [Fe-S] cluster containing proteins such as Ferredoxin (#30 and 31) and Rieske domain protein (#47), which were over expressed in response to Cu depletion. We suggest that overall the electron transport chain was down-regulated, but an impairment of [Fe-S] cluster biosynthesis could be compensated by an up-regulation of *de novo* synthesis of [Fe-S] proteins. This stimulation of [Fe-S] cluster proteins can be linked to the accumulation of Rhodanese family members (#15 to 16), previously described as protecting [Fe-S] clusters [57] in addition to their role in cyanate detoxification.

Previous studies [52], [58] highlighted the transcription factor SPL7 (SQUAMOSA-promoter binding link protein 7) which seems to coordinate the early response to Cu deficiency by targeting a Cu response element (CuRE) in the promoter of genes differentially expressed during Cu deficiency. Unfortunately, we couldn’t performed an analysis on promoters of genes corresponding to the proteins presented in table 3 as *B. napus* genome sequence is not yet available. However, regulation seems to be complex as Cu deficiency induces a down-regulation at transcript level of carbonic anhydrase [52], but it was up-regulated at protein level in our study (#39). It should be noted that the Carbonic anhydrase regulation is independent from SPL7 [52] and thus its regulation should not be directly induced by Cu deficiency. Moreover, previous studies on Cu deficiency also reported a variation of microRNAs [51], [52] or proteases ([59] as well as FTSH2 #14, Table 3 and Figure 5) that could impact on the translation and the proteolysis and thus lead to differences between transcriptomic and proteomic results.

### Crosstalk between Cu, [Fe-S] and Mo

Surprisingly, among all quantified mineral nutrients, only Mo uptake was strongly increased in Cu-depleted plants (Figure 1) and this was further linked to strong up-regulation of *MOT1* gene in roots (Figure 2).

The relation between Mo and Cu homeostasis has been reported several times but remains poorly explained [60]–[63]. As an example, in lesser yam (*Dioscorea esculenta*) [63], molybdenum reached a higher concentration in Cu depleted plants than in plants submitted to any other nutrient deficiencies.

**Figure 6:**
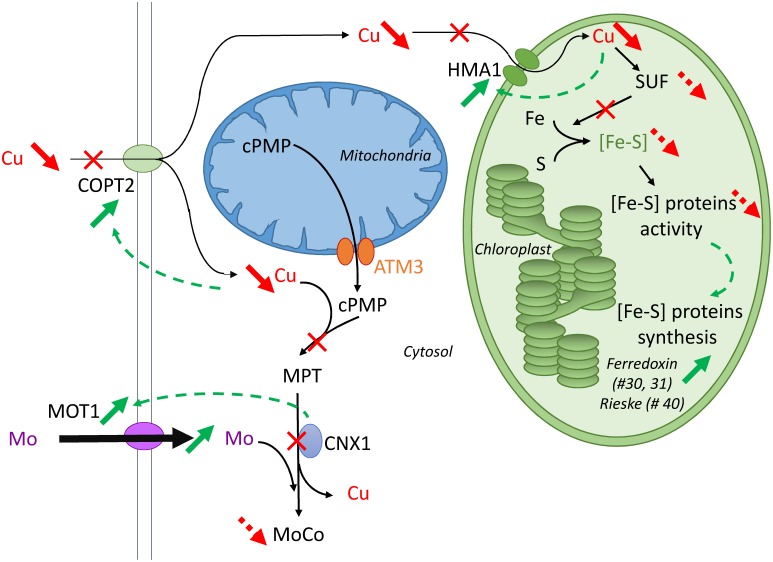
Crosstalk between Cu, [Fe-S] and Mo. Bold arrows indicate variation (green: increase; red: decrease). Full arrows represent observations from this study while dashed arrows have been deduced from the literature. The synthesis of chloroplastic [Fe-S] clusters is controlled by SUF (Sulphur Assimilation Protein), itself controlled by the level of Cu. On the other hand, in the mitochondria, the MoCo precursor cPMP, is exported through mitochondrial membrane by support of the ATM3 transporters. In the cytosol, the second and third steps of MoCo biosynthesis are highly dependent on Cu levels. MoCo is then incorporated into Mo proteins such as Nitrate reductase and into Mo-[Fe-S] proteins such as Xanthine dehydrogenase. The up-regulation of Copt2, HMA1 and Mot1 could be explained by feedback signals due to a low [Cu] in cell, in cytosol and an impairment of CNX1 activity respectively. cPMP: cyclic Pyranopterin MonoPhosphate; MPT: MolybdoPTerin; ATM: ATP-binding cassette Transporters of Mitochondria.

The crosstalk between Cu-Mo and Cu-[Fe-S] established from the literature and the present study are summarised in Figure 6 and can explain the up-regulation of Mo uptake (Figure 1) and expression of [Fe-S] proteins (Rieske #47 and Ferredoxin #30, 31, table 3) by Cu deficiency. Indeed, the chloroplast produces its own [Fe-S] clusters through the SUF (sulfur assimilation proteins) machinery [64], which may be inhibited by Cu deficiency, as previously described in *Bacillus subtilis* [65]. As previously discussed, our leaf proteomic analysis revealed two identified proteins that were over-expressed during Cu deficiency, Ferredoxin and Rieske protein which contain [Fe-S] clusters (#30, 31, 47, Table 3, Figure 6). We may hypothesized that their activity was lowered due to [Fe-S] cluster destabilization by Cu-depletion. This should lead to the observed up-regulation of both proteins to compensate for a possible loss of activity. To minimize the effect of Cu deficiency in chloroplast, the Cu transport across Chloroplast membrane is stimulated through the up regulation of *HMA1* (Figure 2.C, 5, 6).

*MOT1* was previously identified as a S transporter (*SULTR5;2*) and it was identified very recently as a high affinity transporter of Mo [45]. Its localization remains unclear (mitochondrial membrane [46] or plasma membrane [45]). Its precise function has not been reported yet, but its importance for an efficient Mo uptake is acknowledged [66]. At cellular level, Mo is essentially used by the Molybdenum Cofactor (MoCo) which is essential for key enzymes such as nitrate reductase and aldehyde oxidase [66]. The first step of the MoCo biosynthesis occurs in the mitochondria and produces cPMP (Figure 6, [67]). cPMP is then exported across the mitochondria membrane by support of the ATM3 transporter [68]. In the cytosol, it must bind Cu in order to be metabolised into MPT representing a step that should be affected in Cu depleted plants. Then, the CNX1 multidomain protein releases Cu and inserts Mo from MPT to give a functional MoCo molecule [69]. It has been reported that CNX1 binds to the cytoskeleton [70] and could have a role as a Mo sensor, interacting with MOT1 transporter to regulates Mo concentration in the cell [71]. Overall, it would explain how the Cu deficiency indirectly up-regulates the Mo uptake through the increase of MOT1 expression. On the other side, a transcriptomic analysis of *Arabidopsis thaliana* WT and *mot1* mutants submitted to Mo deficiency [72] did not reveal a modification of the expression of genes involved in Cu metabolism.

The impairment of Mo metabolism could also be the cause of Ferredoxin and Rieske up-regulation. Indeed, a strong crosstalk exists between Fe and Me metabolisms: some proteins involved in MoCo biosynethesis are Fe-S (but these Fe-S clusters assembly are independent from the SUF machinery) [66], and Ferredoxin transcripts were up-regulated during an Mo deficiency in *mot1* mutants [72].
